# Mechanical demands of bite in plane head shapes of ant (Hymenoptera: Formicidae) workers

**DOI:** 10.1002/ece3.10162

**Published:** 2023-06-06

**Authors:** Cristian L. Klunk, Marco A. Argenta, Alexandre Casadei‐Ferreira, Marcio R. Pie

**Affiliations:** ^1^ Graduate Program in Ecology and Conservation Universidade Federal do Paraná Curitiba Brazil; ^2^ Department of Civil Construction Universidade Federal do Paraná Curitiba Brazil; ^3^ Biodiversity and Biocomplexity Unit Okinawa Institute of Science and Technology Graduate University Onna Japan; ^4^ Department of Biology Edge Hill University Ormskirk UK; ^5^ Department of Zoology Universidade Federal do Paraná Curitiba Brazil

**Keywords:** biomechanics, finite element analysis, insect, macroevolution, muscular contraction, worker polymorphism

## Abstract

Food processing can exert significant evolutionary pressures on the morphological evolution of animal appendages. The ant genus *Pheidole* displays a remarkable degree of morphological differentiation and task specialization among its workers. Notably, there is considerable variation in head shape within worker subcastes of *Pheidole*, which could affect the stress patterns generated by bite‐related muscle contraction. In this study, we use finite element analysis (FEA) to investigate the effect of the variation in head plane shape in stress patterns, while exploring the morphospace of *Pheidole* worker head shapes. We hypothesize that the plane head shapes of majors are optimized for dealing with stronger bites. Furthermore, we expect that plane head shapes at the edges of each morphospace would exhibit mechanical limitations that prevent further expansion of the occupied morphospace. We vectorized five head shapes for each *Pheidole* worker type located at the center and edges of the corresponding morphospaces. We conducted linear static FEA to analyze the stresses generated by mandibular closing muscle contraction. Our findings indicate that plane head shapes of majors exhibit signs of optimization to deal with stronger bites. Stresses are distinctly directed along the lateral margins of the head, following the direction of muscle contraction, whereas the stresses on the plane head shapes of minors tend to concentrate around the mandibular articulations. However, the comparatively higher stress levels observed on majors' plane head shapes suggest a demand for cuticular reinforcement, like increased cuticle thickness or sculpturing pattern. Our results align with the expectations regarding the main colony tasks performed by each worker subcaste, and we find evidence of biomechanical limitations on extreme plane head shapes for majors and minors.

## Graphical abstract

To assess the role of head shape in stress patterns of *Pheidole* workers, we solve a set of finite element analyses while exploring variation in *Pheidole* worker head morphospace. Our results demonstrate that head shapes of majors show signs of optimization to deal with stronger bites given that stresses dissipate more evenly along the head than in minors. In addition, our results agree with the expectations regarding the main colony tasks performed by each worker subcaste, and we find some evidence of biomechanical limitations on extreme head shapes for majors and minors.

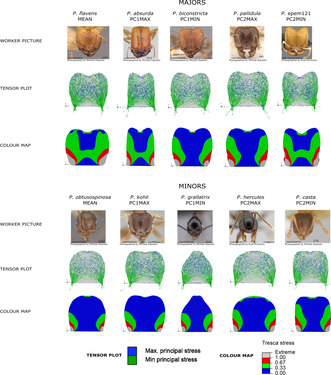

## 1 INTRODUCTION

Food consumption in animals usually involves a phase of mechanical processing, which leads to several morphological adaptations in the involved structures. Among bilateral animals, the mechanical processing of food usually happens with the aid of head structures (Brusca & Brusca, 2003), in a way that the head morphological evolution can be strongly linked to food processing demands. Among insects, which show an incredible species diversity and evolved several modes of food consumption (Krenn, 2019), the biomechanics of food capture and processing seems to be a relevant evolutionary pressure for head and mouth appendices morphological variation (Blanke, Schmitz, et al., 2017; Blanke, Watson, et al., 2017; Blanke et al., 2018; Camargo et al., 2015, 2016; Hörnschemeyer et al., 2013; Weihmann et al., 2015). In some insect lineages, evolutionary pressures lead to the development of strong intraspecific morphological variation. This phenomenon is common among eusocial insects, where individuals of the same colony can have distinct morphologies associated with task specialization, such as food processing (Ferster et al., 2006; Mertl & Traniello, 2009; Wilson, 1953, 2003).

Ants show a reproductive division of labor, in which winged individuals (i.e., queens and males) are specialized in reproduction, and wingless individuals (i.e., workers) execute quotidian colony tasks (Hölldobler & Wilson, 1990; Wilson, 1971). Such division of labor led to substantial morphological adaptations, mainly among workers, especially regarding the loss of flight capacity and the necessity to live on the ground (Galbán et al., 2021; Keller et al., 2014; Liu et al., 2019; Peeters et al., 2020; Peeters & Ito, 2015). Some ant lineages also show morphological variation in the worker caste, a phenomenon called worker polymorphism (Wills et al., 2018; Wilson, 1953). This polymorphism takes on distinct degrees in ant lineages, from continuous changes in worker size to profound morphological modifications that result in discrete worker types (Wilson, 1953). Worker polymorphism potentially enhances the colony division of labor and consequently improves task efficiency (Fjerdingstad & Crozier, 2006; Oster & Wilson, 1978).

In the genus *Pheidole*, workers split into two morphologically discrete subcastes, the minor and major workers (Wilson, 1953, 2003). Minors are small and slender, whereas majors are more robust and have disproportionately larger heads (Friedman et al., 2020; Lillico‐Ouachour et al., 2018; Pie & Traniello, 2007; Rajakumar et al., 2018). While minors are responsible for most of the colony's non‐reproductive tasks, majors usually are associated with specialized responsibilities such as defense and food processing (Mertl & Traniello, 2009; Wilson, 1984, 2003). Workers rely on their mandibles to execute most colony tasks, which involve behaviors such as biting, excavating, carrying, cutting, and fighting (Wheeler, 1910). There is evidence that the multitask use of mandibles influenced the evolution of mandibular morphology in some ant lineages (Zhang et al., 2020). Indeed, the versatility of ant mandibles potentially represents one of the main characteristics of their ecological success (Wilson, 1987). Mandibular movement is driven by two muscle pairs located inside the head: *craniomandibulares externus* (*0md3*) opens the mandibles, whereas *craniomandibulares internus* (*0md1*) is responsible for closing them (Richter et al., 2020; Snodgrass, 1935). *0md1* is the largest muscle of ant workers, occupying up to two‐thirds of the head volume in some species (Paul & Gronenberg, 2002). It connects to the mandibles through a cuticular projection called mandibular apodeme (Richter et al., 2020). The entire muscle constitutes distinct bundles that can differ in fiber type. Some muscular fibers are composed of long sarcomeres optimized to generate strong contractile forces, whereas others are composed of shorter sarcomeres optimized for faster contraction (Gronenberg et al., 1997). These fibers can attach directly to the mandibular apodeme or indirectly through cuticular filaments projected by the mandibular apodeme (Paul & Gronenberg, 1999). As for fiber and attachment types, the arrangement of the *0md1* bundles can vary inter and intraspecifically. However, the relative position of each bundle inside the head capsule is more conserved, and there is evidence that the simultaneous contraction of all *0md1* fibers optimizes forceful movements (Paul & Gronenberg, 2002). This complexity of *0md1* makes it the key to the versatility of ant mandible movements. Moreover, evolutionary pressures toward an increase in muscle volume can influence the evolution of worker head size and shape (Paul & Gronenberg, 1999).

Size might be the primary driver of morphological differentiation between *Pheidole* worker subcastes (Pie & Traniello, 2007), potentially evolving at higher rates than shape (Economo et al., 2015; Friedman et al., 2019; Pie & Tschá, 2013). However, there is evidence that the head shape of worker subcastes evolved more independently from each other than the mesosoma shape (Friedman et al., 2020). The specialized roles of majors in food processing suggest that diet can be a driver of *Pheidole* worker morphological evolution, mainly due to the necessity to process hard food items (Casadei‐Ferreira et al., 2021; Holley et al., 2016). Several *Pheidole* species add seeds to their diet (Moreau, 2008; Rosumek, 2017), whose consumption usually requires powerful bites, improved by an increase in muscle volume and, consequently, the head size of majors (Lillico‐Ouachour et al., 2018; Paul & Gronenberg, 1999). Although recent attempts suggest that the morphological variation between *Pheidole* species in head shape and size seems not to be related to differences in diet (Casadei‐Ferreira et al., 2021; Holley et al., 2016), little is known about how the head shape is associated with the mechanical demands of biting, which differ between worker subcastes. Recent works suggest that even slight morphological modifications in *Pheidole* worker mandibles could lead to differences in bite performance between worker subcastes and species, according to the primary roles of each worker subcaste in the colony (Huang, 2012; Klunk et al., 2021), as also observed in other ants (Larabee et al., 2018).

Given the prominence of *0md1* in ant workers, we can expect that muscle contraction exerts significant mechanical demands on the worker's head capsule, with patterns of stress generated by the *0md1* contraction potentially varying according to head shape, which could effectively be tested with a biomechanical simulation. We aimed to investigate the relationship between head shape and biomechanical performance by finite element analysis (FEA) (Kupczik, 2008; Rayfield, 2007) in *Pheidole* worker plane head shapes. Although a simplistic perspective, the use of 2D data in biomechanical simulations proved to be effective as a first approximation to the mechanical demands of complex structures (Marcé‐Nogué et al., 2013), as demonstrated for the effects of biting behavior in crocodilians (Pierce et al., 2008, 2009), theropods (Rayfield, 2005) and *Tyrannosaurus rex* (Rayfield, 2004) skulls, as well as bite loading in vertebrate jaws (Deakin et al., 2022) and the mechanical consequences of borrowing on the trilobite cephalic region (Esteve et al., 2021).

Here we considered *Pheidole* species that represent the main variation in worker plane head shapes based on previously published morphospaces of major and minor *Pheidole* workers (Casadei‐Ferreira et al., 2022). In doing so, we avoid a more subjective choice of taxa and maximize morphological variation (Tseng, 2021). Many of those species were included in the most recent phylogeny of the genus, showing a varied degree of phylogenetic relatedness (Economo et al., 2015), representing lineages from the Neotropics, Australasia, Africa, and Madagascar. Of the 10 *Pheidole* species here considered, six were present in that phylogeny and diverged very anciently in *Pheidole*'s diversification history (Economo et al., 2015).

Our intention was to investigate the mechanical behavior of idealized plane head shapes while exploring the morphospace limits of *Pheidole* workers. We recognize that some morphological characteristics of the head disappear under such an approach, like the effects of the variation in head cuticle thickness. However, with a plane stress approach, we can isolate the influence of the head outline on the variation in stress patterns (stress magnitude, direction, and type). We aimed to investigate how the mean plane head shapes of majors and minors—which are good representatives of the most common head shapes observed among *Pheidole* workers—differ in stress patterns and if plane head shapes located at the extremes of the occupied morphospaces show stress patterns that suggest some mechanical constraints, which potentially could explain the low frequency of such plane shapes in extant *Pheidole* lineages. We hypothesize that the mean plane head shape of *Pheidole* major and minor workers will display substantial differences in stress patterns, with majors showing patterns associated with the capacity to deal with stronger bite forces than minors. In addition, we expect that the plane shapes located on the morphospaces' extremes will show distinct stress patterns from the mean plane shapes, with signs of mechanical limitations that prevent its higher frequency on current *Pheidole* lineages. Given that the amount of head cuticle that shows any sculpturing pattern varies intra and interspecifically among *Pheidole* species (*personal observations*), we also tested if such variation is associated with stress patterns generated by the *0md1* contraction, hypothesizing that majors have an increased area of the head dorsal wall covered with sculptures than minors.

## 2 MATERIALS AND METHODS

### 2.1 Head shapes

We chose *Pheidole* species with particularly extreme morphologies by exploring the morphospace inferred by Casadei‐Ferreira et al. (2022) using 2D geometric morphometrics data. The plane axis considered by Casadei‐Ferreira et al. (2022) contemplates variation in head height and width, two of the main characteristics associated with the morphological variation among *Pheidole* worker heads (Casadei‐Ferreira et al., 2021). We selected *Pheidole* species close to the edges of the first two principal component analysis (PCA) axes and the mean shape for each subcaste (Casadei‐Ferreira et al., 2022), totaling 10 species (five for each subcaste, Table 1). We vectorized head shapes using Inkscape® based on images of specimens available on AntWeb (2021) and exported those vectors as OBJ files using Blender 2.83®. With Fusion 360 (AUTODESK), SAT geometries were generated using the OBJ files and imported into the finite element solver Abaqus 6 (Dassault Systèmes).

**TABLE 1:** Main characteristics of the simulated plane head shape models of *Pheidole* workers, including species, subcaste type, position in the morphospace, undeformed mesh area, number of elements, load magnitude, and mean Tresca equivalent stress value after simulation.

Species	Subcaste	Morphospace position	Mesh area (mm^2^)	Number of mesh elements	Applied load (N)^a^	MTESV (N/mm^2^)^b^
*Pheidole absurda*	Major	PC1 max	3.510	82,981	1.00	0.78
*Pheidole biconstricta*	Major	PC1 min	1.710	82,700	0.70	0.67
*Pheidole pallidula*	Major	PC2 max	1.410	80,750	0.63	0.67
*Pheidole* epem121	Major	PC2 min	1.070	81,912	0.55	0.71
*Pheidole flavens*	Major	Mean	0.665	81,745	0.44	0.68
*Pheidole kohli*	Minor	PC1 max	0.951	85,269	0.52	0.65
*Pheidole grallatrix*	Minor	PC1 min	0.225	82,408	0.25	0.71
*Pheidole hercules*	Minor	PC2 max	0.556	82,363	0.40	0.67
*Pheidole casta*	Minor	PC2 min	0.141	84,075	0.20	0.69
*Pheidole obtusospinosa*	Minor	Mean	0.337	82,784	0.31	0.69

^a^ Load applied at each head side.

^b^ Mean Tresca equivalent stress value.

### 2.2 Finite element analysis

We apply unitary and constant thickness to head plane models to define plane stress analysis. Such a procedure considers that the modeled structure has two main dimensions, and the stresses in the third dimension are negligible. The finite element meshes were designed with plane triangular quadratic elements (CPS6M). We define the mesh density after a mesh convergence procedure of three plane head shapes (i.e., *Pheidole flavens*, *Pheidole grallatrix*, and *Pheidole obtusospinosa*), which are good representatives of the morphological variation of the plane head shapes here considered. We defined more simplistic loading and boundary conditions to perform the mesh convergence tests and analyzed the variation in Tresca equivalent nodal stress values with changes in mesh density. Once the error between the current and last mesh densities in nodal Tresca stress achieved <2% in three different nodes, we chose the coarser mesh of the converged pair to represent the final mesh density. Mesh convergence was achieved at the same density in all three plane head models (Table S4), so we applied the defined mesh density to all plane head representations (Table 1). We determined the material properties according to data available in the literature. Therefore, we designated Young's modulus as 2.75 GPa (Brito et al., 2017) and the Poisson ratio as 0.3 (Klunk et al., 2021; Larabee et al., 2018; Wang et al., 2022; Zhang et al., 2020). We determined the cuticle as an isotropic and linear elastic material and applied the same homogeneous material properties for each head plane model.

**FIGURE 1:**
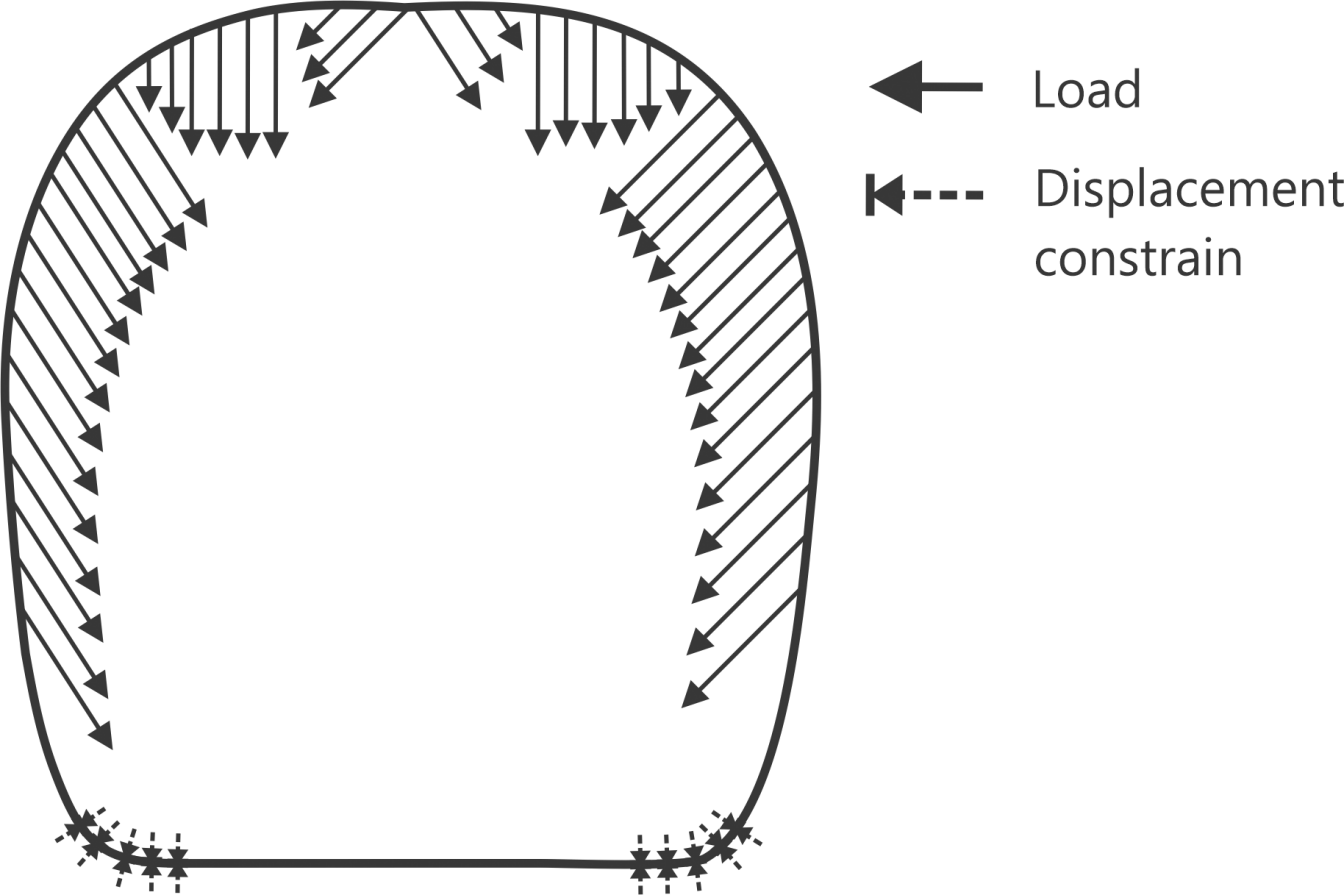
Diagram depicting the boundary conditions of *Pheidole* worker finite element analysis simulations. The same load magnitude was applied to each head side. Nodal displacement constraints were applied on the same number of nodes in each head side and simulation and were positioned to approximate the mandible articulation with the head. Inclined load vectors were defined with an inclination of 45°.

To simulate the loads generated by the contraction of mandibular closing muscles, we applied normal loads on the nodes of each head side (Figure 1), approximating the pennation angle of *0md1* usually observed in ant workers (Boudinot et al., 2021; Lillico‐Ouachour et al., 2018; Paul, 2001; Paul & Gronenberg, 2002; Püffel et al., 2021; Richter et al., 2019, 2020, 2021) that maximizes force generation, as suggested for leaf‐cutting ants (Püffel et al., 2021). We applied a 1 N load on each head side to the model with the largest surface area and normalized the load of the remaining models according to the difference in surface area from the reference model (Table 1; Marcé‐Nogué et al., 2013). We fixed nodal displacement to zero in all directions in the corners of the head base. This procedure approximates the positioning of the mandibles to simulate their reaction forces during a bite. We fixed the same number of nodes in each head side and model. We performed one linear static simulation for each head shape.

**FIGURE 2:**
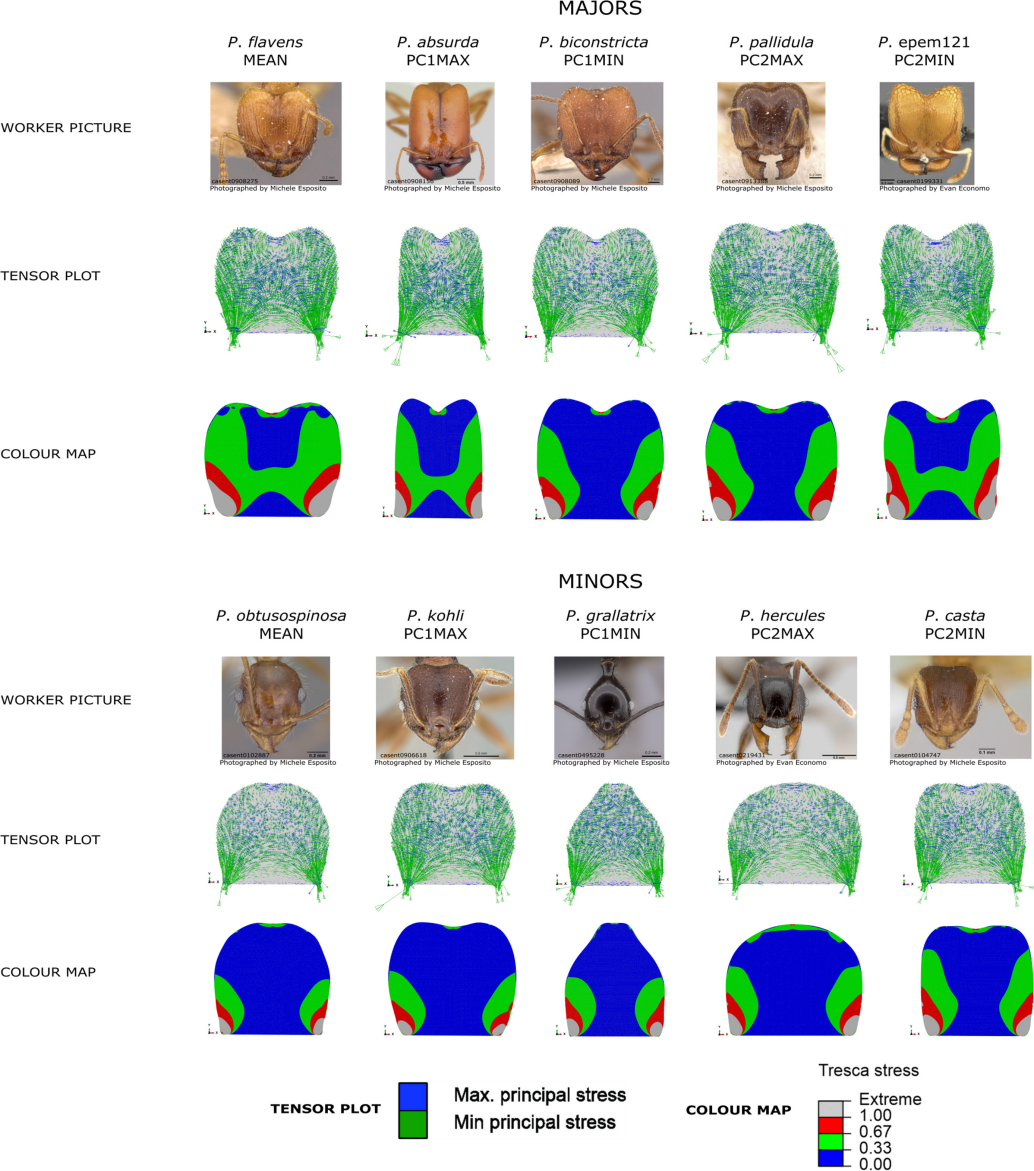
Tensor plots and color maps depicting the results of finite element analysis simulations in *Pheidole* worker 2D heads. The first row of each panel shows the picture used to generate the head shape vectors, as available on AntWeb (AntWeb, 2021). The second row depicts tensor plots depicting the distribution of principal stresses. The third row shows the color maps, which depict the variation of proportional stress levels based on the Tresca failure criterion. Tensor plots and color maps have stress limits normalized based on a reference model, and the results for each head shape can be directly compared.

To visualize the resulting stress patterns, we used tensor plots of principal stresses (Figure 2). Principal stresses are normal stresses that occur at plane orientations where sheer stress is zero, represented by minimum and maximum principal stresses (Hibbeler, 2016). Arrows indicate the direction (positive or negative along the x and y axes), normalized magnitude (arrow size), and stress type (compression or tensile, according to the orientation of the arrowheads). Green arrows depict the minimum principal stresses and represent here essentially compressive stresses (arrowheads pointing inward; Figure 2). Blue arrows, otherwise, represent the maximum principal stresses, which range here from compressive to tensile stresses (arrowheads pointing outward; Figure 2). To have a more comprehensive visualization of the distribution of stress levels along *Pheidole* worker plane heads, we also used color maps depicting stress variation in normalized magnitude, based on a failure criterion. We can represent stress values from FEA under several stress variables, being the better choices related to the structure material behavior. In a state of plane stress, as simulated here, any element is subjected to two normal and one shear stress (Hibbeler, 2016). To reliably consider the material strength and its resistance to failure, such combined sources of stress are reduced to a single stress component by applying a stress transformation that follows a specific failure criterion. Here we considered the Tresca failure criterion, which is more conservative (Özkaya et al., 2017). Tresca failure criterion assumes that material failure happens due to shear stress, and the direction of principal stresses observed on our tensor plots (Figure 2) justifies the adoption of this criterion. We scaled the stress range of each simulation based on the maximum stress value of a reference model to improve visualization and allow comparison between species. Therefore, the non‐normalized stress values of each simulation are meaningless in those plots, and we interpreted the qualitative differences between simulations as representing proportional differences in stress distribution.

### 2.3 Statistical analyses

We applied the intervals method (Marcé‐Nogué et al., 2017) to evaluate how plane head shapes differ in the area covered by distinct ranges of stress values. This method defines comparable stress intervals from FEA results and calculates the area covered by each stress interval based on the sum of the element area. From a predefined upper threshold of stress, which determines the highest stress interval in the dataset and is a reference for the generation of the remaining stress intervals, the method can generate any number of stress intervals, sum up the area occupied by those intervals on each model, and calculate the proportion of area covered by each interval for all models. These area proportions are new variables that can be used in further statistical tests or ordination analyses, such as PCAs (Marcé‐Nogué et al., 2017).

To apply this method, we extracted values of stress and area from the elements of each plane head model and removed the elements representing the 2% highest stress values for each simulation since they usually represent artificially high values (Marcé‐Nogué et al., 2016, 2017). We transformed the stress values to their natural logarithm to avoid the influence of outliers. We defined the upper threshold value as 0.69, to characterize the highest stress interval with only 2% of the highest stress values from all simulations (Marcé‐Nogué et al., 2017). We generated datasets with distinct intervals (5, 15, 25, 50, and 75) and performed a PCA with each dataset to define the ideal number of stress intervals. We considered the scores of PC1 and PC2 of each dataset as variables in linear regressions with the scores of equivalent PCs of the next stress interval (e.g., PC1_5intervals_ ~ PC1_15intervals_), considering the coefficient of determination (*R* ^2^) to analyze the convergence of PC scores (Marcé‐Nogué et al., 2017). When *R* ^2^ ceased to increase, the number of intervals of the dataset where *R* ^2^ converged was considered the final number of intervals (Marcé‐Nogué et al., 2017). Convergence occurred within 15 intervals. Therefore, we conducted the PCA with this dataset to investigate how plane head shapes differ in the proportion of head area covered by the distinct intervals of stress. We performed all statistical analyses in R version 4.1.3 (R Core Team, 2022). We also tested if the plane head shapes vary in the magnitude of Tresca equivalent stress values through a Kruskall–Wallis test and post hoc Dunn tests with Bonferroni corrections for repeated tests.

Finally, we tested whether *Pheidole* workers differ in the sculpturing pattern of their heads according to the distinct stress distributions observed in the tensor and color plots. We defined four categories of cuticle sculpturing: category 0 represents species with an overall smooth head cuticle, showing some sculpture around the antennal insertion; category 1 represents species with some sculpturing around the regions of mandibular articulation; category 2 denotes species with additional sculpture spreading toward the head lateral margins; and category 3 stands for species whose most head dorsal cuticle is sculptured. We classify sculpturing patterns of 143 *Pheidole* species considered in Casadei‐Ferreira et al. (2022) for the head shape morphospace of *Pheidole* species. We obtained worker full‐face pictures from an online repository (AntWeb, 2021). Whenever more than one picture for each worker type was available, and there was variation in sculpture patterning, we classified the worker according to the highest category detected, prioritizing the patterns found on type specimens. Then, we tested if *Pheidole* workers differ in head sculpturing pattern among those four categories through a chi‐squared test, with a post‐hoc test with Bonferroni correction to test for pairwise differences between sculpture categories, using the R package *chisq.posthoc.test* (Ebbert, 2019).

We used the R package *dplyr* version 1.0.9 to manipulate the data (Wickham et al., 2022), *FactoMineR* version 2.4 (Lê et al., 2008), and *factoextra* version 1.0.7.999 (Kassambara & Mundt, 2020) to perform the PCA, and *ggplot2* version 3.3.5 (Wickham, 2016), *ggpubr* version 0.4.0 (Kassambara, 2020), *viridis* version 0.6.2 (Garnier et al., 2021), *tidyverse* version 1.3.1 (Wickham et al., 2019), and *hrbrthemes* version 0.8.0 (Rudis, 2020) to generate plots. R code (Data S1), stress (Data S2), and area (Data S3) data regarding the application of the intervals method, as well as the data of *Pheidole* species sculpturing pattern (Data S4) are available as Supplementary Files.

## 3 RESULTS

### 3.1 Tensor plots

The plane head shape of majors and minors showed differences in stress patterns. *P. flavens*, which approximates the mean shape of majors, showed proportionally higher levels of stress spreading throughout a larger area of the head, mainly along its lateral margins following the direction of muscle contraction (Figure 2). On the other hand, the mean shape of minors, represented by *P*. *obtusospinosa*, showed a concentration of stress around the regions of mandibular articulation, but proportionally lower stress levels distributed throughout the remaining of the head (Figure 2). Among majors, plane head shape varies more subtly in stress patterns across the morphospace than in minors. *Pheidole absurda* (PC1max) and *P*. epem121 (PC2min) showed a stress pattern similar to that of the mean shape, whereas *Pheidole biconstricta* (PC1min) and *Pheidole pallidula* (PC2max) exhibited slightly reduced stress levels along the head, especially on the lateral margins (Figure 2). Minors have a higher morphological variation along the morphospace, although their stress patterns are also similar in general, except that *Pheidole hercules* (PC2max) showed a considerable amount of stresses along the posterior margin of the head, and *Pheidole casta* showed proportionally higher stresses along the lateral margins of the head (Figure 2). In a thinner analysis considering tensor plots, *P*. *hercules* draws attention due to a reduced concentration of stress on the head center than the remaining shapes (Figure 2), which is possibly related to its wider head, leading to significant dissipation before stresses achieve the central area of the head. In *P*. *grallatrix* (PC1min), which has a narrower head, an opposite pattern was observed, with a denser concentration of stress on the central head region, especially of compressive stresses along the x‐axis (lateral compression) (Figure 2). *P. casta* (PC2min) and *P*. *kohli* (PC1max) showed similar stress patterns, having in general higher stress levels than *P*. *hercules* and *P*. *obtusospinosa*, and proportionally higher levels of vertical compressive stresses along the lateral margins than *P*. *grallatrix*, although at substantially lower levels than what happens in majors (Figure 2).

### 3.2 Intervals method

**FIGURE 3:**
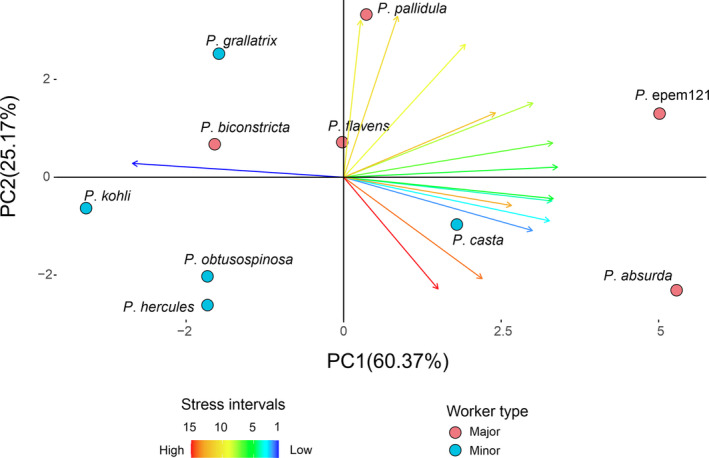
Principal component analysis (PCA) plot showing the differences between *Pheidole* head shapes in the amount of area covered by each of the 15 stress intervals, considering the first two PCA components (see text for details). Stress intervals ranged from 1 (lowest stress levels) to 15 (highest stress levels).

To evaluate quantitatively the proportion of head area covered with different stress intervals, we used a PCA whose input variables were the proportional amount of area covered by each of the 15 intervals of stress. The first two components explained 85.54% of the variance and were considered here for further discussion. PC1 explained 60.37% of the variance and split species with proportionally larger areas of the head covered by intervals of intermediate stress levels (i.e., *P*. *absurda* and *P*. epem121) from species with a larger area covered by the lowest stress interval (i.e., *P*. *kohli*; Table S1). Interestingly, the positive range of PC1 is occupied predominantly by plane head shapes of majors, except for the presence of *P*. *casta*. In contrast, the PC1 negative spectrum depicts mostly minor worker plane heads, except for *P*. *biconstricta*. The head shape of *P*. *flavens* lies on the origin (Figure 3), suggesting a more homogeneous distribution of stress intervals. PC2 explained 25.17% of the variance and is positively associated with intervals 10–11 (Table S1). This component mainly separates *P*. *pallidula* and *P*. *grallatrix* from the remaining species in its positive range (Figure 3).

### 3.3 Tresca equivalent stress values

Non‐normalized stress levels based on Tresca failure criteria differed significantly between *Pheidole* plane head shapes after removing from each model their 2% highest stress values (Kruskall–Wallis ${\chi^{2}}_{9}$ = 10,547; *p* < .001). *P. absurda* showed the highest mean stress value among all shapes (0.78 N/mm^2^), whereas *P*. *kohli* showed the lowest mean stress (0.65 N/mm^2^; Table 1; Figure S1). Only two species pairs showed no difference in mean stress levels after post hoc Dunn tests with Bonferroni corrections for repeated tests, which were *P*. *flavens* versus *P*. *hercules* and *P*. *biconstricta* versus *P*. *pallidula* (Table S2). Although statistically different, the mean non‐normalized stress values of *P*. *flavens* (0.68) was only slightly lower than for *P*. *obtusospinosa* (0.69) (Table 1; Figure S1).

### 3.4 Cuticle sculpturing pattern

*Pheidole* workers differed in the amount of sculpture covering the dorsal head cuticle (χ^2^ = 46.83, df = 3, *p* = 3.778e‐10). Specifically, minors of *Pheidole* species tended to have sculpturing patterns of categories 0 and 1 (small regions of the head sculptured), and majors patterns of category 3 (almost the complete dorsal area of the head covered with some sculpture), whereas there was no difference regarding category 2 (Table S3; Figure S2).

## 4 DISCUSSION

Our FEA simulations demonstrated that plane head shape variation affects stress patterns due to *0md1* contraction. Tensor plots exposed substantial distinctions in stress patterns between the most common worker plane head shapes (i.e., the mean shapes). The head shape of *P*. *flavens* showed a propensity to dissipate stresses along a larger area of the head than the head shape of *P*. *obtusospinosa*, where stresses tended to concentrate more around the regions of mandibular articulation. Interestingly, such head shapes showed only a slight difference in the mean non‐normalized stress value, with *P*. *obtusospinosa* having a slightly higher value than *P*. *flavens*. Such differences involving the most common head shapes observed among current *Pheidole* lineages suggest that majors are more suited to dissipate stresses and avoid stress concentration, being able to withstand higher forces related to bite loading when no other morphological aspects are being taken into account (e.g., cuticle thickness or variation in material properties).

Regarding the PCA based on the intervals method, the mean plane head shapes do not represent the most distant pair on PC1, with the head shape of *P*. *flavens* showing a more homogeneous distribution of stress intervals than *P*. *obtusospinosa*, which showed a larger area of the head covered with intervals of low stress. On PC2, their separation is more evident, with *P*. *flavens* showing a larger area of the head covered with intervals of intermediate stress levels when compared to *P*. *obtusospinosa*, which showed a slightly higher amount of head area covered by the highest stress interval, which is located around the regions of mandibular articulation. Most major heads have a higher coverage of high‐stress intervals than minor worker heads, agreeing with the patterns observed on tensor plots and color maps, which suggest a more favorable stress dissipation in major worker plane head shapes. The plane head shapes of *P*. epem121 and *P*. *absurda* are particularly distinct because they depart even more from the remaining plane shapes regarding the percentage of area covered with intermediate toward high‐stress values. These shapes also showed higher mean values of Tresca equivalent stresses. *P. absurda* and *P*. epem121 represent plane head shapes located in more isolated regions of their morphospace (Casadei‐Ferreira et al., 2022), which means that few *Pheidole* species explored such head contours, drawing attention to the possibility of biomechanical limitations related to high‐stress generation on these head shapes that prevent their widespread among current lineages. On the other end of PC1, the plane head shape of *P*. *kohli* departs from the remaining head shapes by showing a larger area covered by the interval of lowest stress, reinforcing its tendency, observed on the tensor plot, to heavily concentrate stresses around the regions of mandibular articulation. This mechanical behavior may help to explain why the plane head shape of *P*. *kohli* is poorly explored in current *Pheidole* lineages (Casadei‐Ferreira et al., 2022).

Head shape evolution is crucially associated with insect bite forces (Blanke et al., 2018; Blanke, Watson, et al., 2017). In general, there is a strong correlation between head width and bite force capacity in insects (Blanke, 2019; Rühr et al., 2022) since the generation of forceful bites demands an increase in mandibular closing muscle volume, which in turn requires wider heads to accommodate such larger muscles (Paul, 2001; Paul & Gronenberg, 2002; Püffel et al., 2021, 2022). In ants, *0md1* occupies a significant volume of the worker head (Boudinot et al., 2021; Khalife et al., 2018; Lillico‐Ouachour et al., 2018; Paul & Gronenberg, 2002; Richter et al., 2019, 2020, 2021). Our results suggest that the plane head shape of majors and minors partially reflects the mechanical demands of bite associated with their main colony tasks. *Pheidole* majors typically have broader and heart‐shaped heads that dissipate the stresses generated by the contraction of *0md1* along a wider plane head area, avoiding stress concentration and hence improving its capacity to deal with stronger bites. Minors, otherwise, usually have narrower and round‐shaped heads that show more constrained stress spread, with a tendency to concentrate stresses on the regions of mandibular articulation. Associations between head shape and bite force in ant workers are not novel. Several authors already suggested that broader and heart‐shaped heads can harbor a proportionally larger volume of muscle fibers than elongated heads, consequently generating stronger bites, whereas prolonged head shapes tend to be associated with a distribution of muscle fibers that benefit faster contractions (Khalife et al., 2018; Paul, 2001; Paul & Gronenberg, 2002; Püffel et al., 2021; Richter et al., 2023). The importance of bite force in *Pheidole* majors is clearly illustrated by the fact that *0md1* increases disproportionately in relation to minors, at the expense of other tissues of the worker's head, such as the central nervous system (Lillico‐Ouachour et al., 2018). Our results, however, highlight that the morphological distinctions between head shapes go beyond their differences in the capacity to store muscle fibers and rely on idiosyncrasies in the responses to the mechanical demands of bite loading, as also suggested for *Melissotarsus* Emery 1877 ants (Khalife et al., 2018).

Broadening of the head is characteristic of some specialized ant worker subcastes, as happens in *Pheidole* (Lillico‐Ouachour et al., 2018; Pie & Traniello, 2007; Pie & Tschá, 2013), *Atta* (Püffel et al., 2021), *Cephalotes* (Powell, 2008), *Solenopsis* and *Pogonomyrmex* (Ferster et al., 2006), among others. Morphological specialization in the ant worker caste is usually associated with some degree of task specialization (Oster & Wilson, 1978; Wills et al., 2018). In *Pheidole*, the division of labor strongly correlates with the morphological differences between worker subcastes. Majors are mainly recruited for food processing and defense (Mertl & Traniello, 2009; Wilson, 1984), behaviors that demand stronger and long‐standing bites, like crushing food items. Minors lead the remaining non‐reproductive colony tasks, such as brood care, foraging, and colony maintenance (Mertl & Traniello, 2009; Wilson, 1984), which demand a more generalist use of the mandibles, including the generation of faster, subtler, and more repeatedly mandibular movements.

Besides the main morphological differences between *Pheidole* workers, there is substantial interspecific variation in worker morphology that potentially reflects ecological specializations. The need to capture and process food can considerably affect morphological evolution. In ants, such demands will reflect mainly on the morphological evolution of the worker head and mandibles (Barden et al., 2020; Booher et al., 2021; Ohkawara et al., 2017; Powell & Franks, 2005, 2006). In *Pheidole*, the fact that several lineages add seeds to their diets (Moreau, 2008; Rosumek, 2017) leads to the hypothesis that seed consumption could have been a significant evolutionary pressure toward the evolution of major workers, and to explain the interspecific morphological diversity of the genus (Moreau, 2008). However, investigations so far did not reveal a meaningful relationship between the consumption of seeds and head size (Holley et al., 2016) and shape (Casadei‐Ferreira et al., 2021). Our results showed that plane head shapes of majors did not differ substantially in stress patterns. Although *P. absurda* is the only species here considered known to consume seeds, it did not show a stress pattern considerably distinct that could suggest a better capacity to withstand stronger bites than the remaining head shapes. However, it showed the higher mean Tresca equivalent stress value among all plane head shapes, indicating that such a plane shape is subjected to relatively higher reaction forces from the mandibles. Together, those results agree with the fact that regardless of the consumption or not of seeds, majors generally are faced with tasks that demand stronger bites (Mertl & Traniello, 2009; Wilson, 1984), as defense or even the processing of other hard food items, like arthropod exoskeletons. Therefore, the ecological demands associated with *Pheidole* majors seem to result in a morphological convergence regarding their head plane shape. Minors, otherwise, are faced with a more complex and generalist set of colony tasks (Mertl & Traniello, 2009; Wilson, 1984), being submitted to a more diverse array of evolutionary pressures, potentially leading to the evolution of head shapes that depart more from the general stress pattern of the standard minor plane head shape, as observed on the tensor plots. Current evidence suggests that the geographic distribution of *Pheidole* species is strongly associated with worker head shape evolution (Casadei‐Ferreira et al., 2022). In contrast, nesting habit seems to have the potential to limit the variation of major workers' head size (Mertl et al., 2010).

Majors of *Pheidole* showed an increased area of the head dorsal cuticle covered with some sculpturing pattern, which agrees with our hypothesis that majors have, in general, a sculpturing pattern that follows the distribution of stresses observed in their plane head shapes. Increased stress levels could require some cuticular reinforcement for such mechanical demands. More sculptured cuticles tend to be thicker (Buxton et al., 2021), but even cuticles with the same thickness could potentially differ in their mechanical response when covered with distinct levels of surface roughness (Hellenbrand, 2022). There is evidence that predatory ants, which may need to withstand higher mechanical demands to capture their prey, are more sculptured than non‐predatory species (Gibb et al., 2015). However, cuticular sculpturing patterns can be associated with several distinct functions. The occurrence of sculptures is highly variable along the ant phylogeny (Hellenbrand, 2022), so more effort is needed to investigate the role of cuticular sculpturing and mechanical resistance.

Our results reinforce the idea that evolutionary pressures in the morphological evolution of *Pheidole* workers seem to act independently between worker types (Friedman et al., 2020; Pie & Traniello, 2007) due to distinct mechanical demands associated with their differential roles in the colony. *Pheidole* evolution has been investigated extensively, yet many aspects of its morphological diversity and worker dimorphism remain unexplored. Here we demonstrated that differences between the plane head shape of major and minor workers have profound implications for the stress patterns generated by the contraction of the mandibular closing muscles, agreeing with the suggestion that head shapes in *Pheidole* workers evolved more independently than other body regions (Friedman et al., 2020). In addition, the plane head shape of majors differs less interspecifically than those of minors in stress patterns, suggesting that task specialization between *Pheidole* workers leads to a degree of convergence in head shape, especially leading to the evolution of broader and more heart‐shaped heads in majors. Interestingly, some species we investigated here have both worker types with extreme plane head shapes. Namely, the minor workers of *P*. *casta*, *P*. *grallatrix*, and *P*. *obtusospinosa*, here considered for FEA, have major workers with head shapes near to the extreme head shapes represented here by *P*. *absurda*, *P*. epem121, and *P*. *biconstricta*, respectively. Among the majors here considered, *P*. epem121 has a minor worker whose head shape resembles the extreme head shape of *P*. *kohli* (Figure S3).

Our results indicate that, in general, the plane head shapes of majors have a better capacity to dissipate stresses along the head than minors, which tend to concentrate stresses on a small head area. Since no differences in cuticle thickness were being considered under our approach, which could justify the occurrence of stress concentration in thicker cuticle regions, such distinctions in stress patterns suggest that plane head shapes of majors can withstand stronger bites by dissipating stresses along a larger area of the head. Supporting this mechanism, we showed that the head shapes of majors usually have a considerably more sculptured dorsal surface, which increases the thickness of the cuticle and, hence, could improve the capacity to deal with higher stresses. Further studies are required to investigate in more detail the role of *Pheidole* worker head shape in bite mechanical performance, especially considering the three‐dimensional morphology of this structure. This way, relevant aspects of the head morphology can be addressed, such as the role of the endoskeleton (Blanke et al., 2018; Boudinot et al., 2021; Kubota et al., 2019; Richter et al., 2019, 2020, 2021) and cuticle thickness on stress distribution. In addition, the influence of cuticular sculpturing on the mechanical response to loading remains to be further tested (Buxton et al., 2021; Hellenbrand, 2022). Despite being a more simplistic approach, applying FEA on plane structures proved to be a valuable tool to reveal differences in stress patterns between the head shapes of *Pheidole* workers. We suggest a more widespread application of this approach in other animal lineages for instances where 3D structures are too complex to allow the simultaneous investigation of many species (Esteve et al., 2021; Fletcher et al., 2010; Pierce et al., 2008, 2009; Rayfield, 2004, 2005).

## AUTHOR CONTRIBUTIONS

**Cristian L. Klunk:** Conceptualization (equal); formal analysis (lead); investigation (equal); methodology (equal); writing – original draft (lead); writing – review and editing (lead). **Marco A. Argenta:** Formal analysis (equal); methodology (equal); supervision (equal); writing – original draft (equal); writing – review and editing (equal). **Alexandre Casadei‐Ferreira:** Conceptualization (equal); data curation (equal); writing – original draft (equal); writing – review and editing (equal). **Marcio R. Pie:** Conceptualization (equal); supervision (equal); writing – original draft (lead); writing – review and editing (lead).

## CONFLICT OF INTEREST STATEMENT

We declare we have no competing interests.

## Supporting information


Figures S1–S3
Tables S1–S4

Data S1

Data S2

Data S3

Data S4

## Data Availability

R code (Data S1) regarding the application of the intervals method, stress (Data S2), and area (Data S3) data of each element and head shape, as well as the classification of *Pheidole* species regarding their sculpturing pattern (Data S4), are available as Supplementary Files.
