# Supplementary material for: Mechanical demands of bite in plane head shapes of ant (Hymenoptera: Formicidae) workers
Source: Ecol Evol. 2023 Jun 6;13(6):e10162. doi: 10.1002/ece3.10162 (PMC10244895; doi:10.1002/ece3.10162)
Supplement: Supplementary file 1 — Figures S1–S3 Tables S1–S4 [file ECE3-13-e10162-s003.docx]

Appendix

**File S1.** R code for the statistical analysis.

**File S2.** Raw stress data from FEA of each species.

**File S3.** Raw area data from model meshes of each species.

**File S4.** Data of sculpturing patterns in *Pheidole* worker head cuticle.


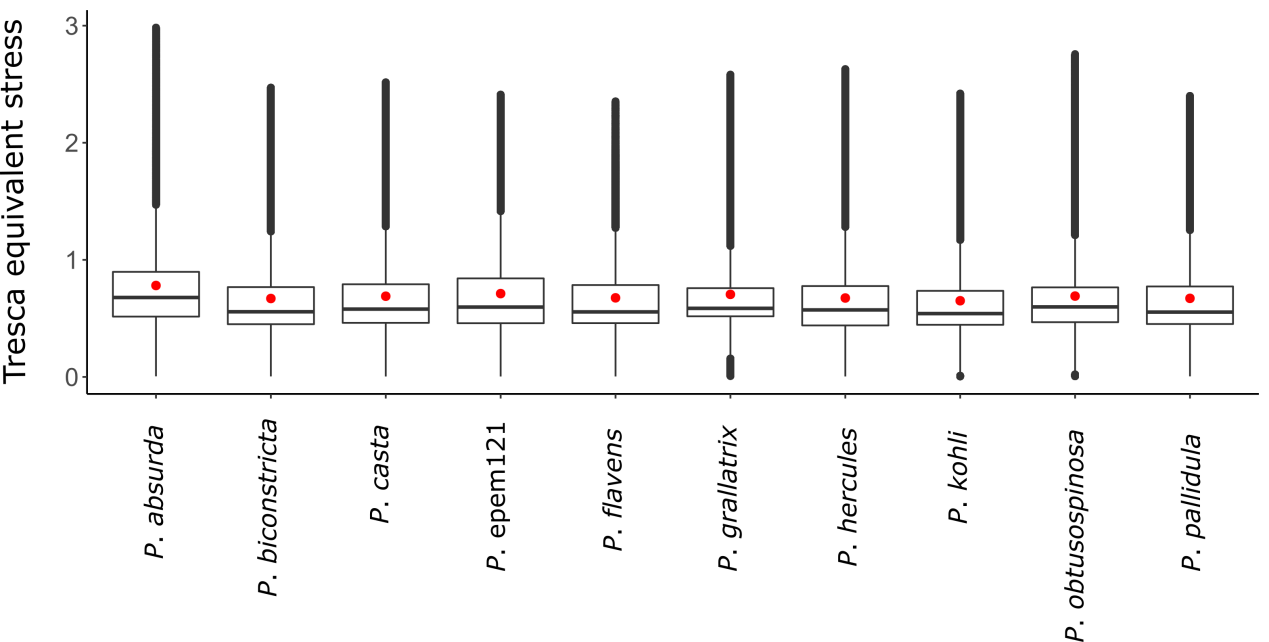


**Fig.S1.** Stress values of each head shape, based on Tresca failure criterion. 2% of higher stress values of each head shape were removed.


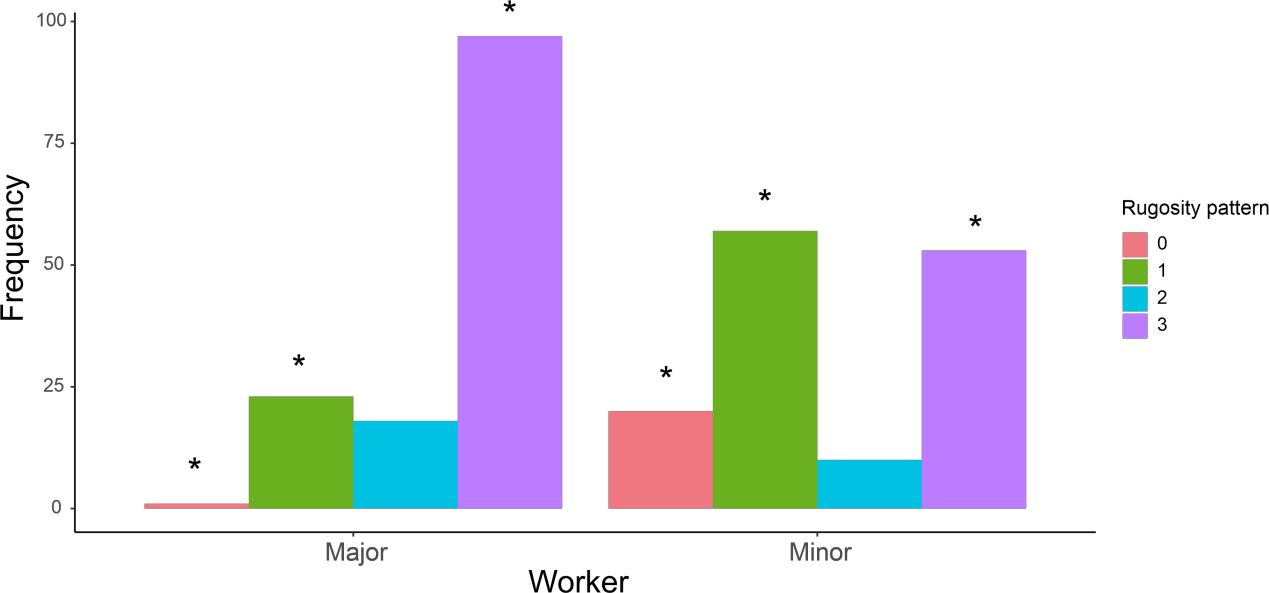


**Fig.S2.** Barplot depicting the frequency of cuticle rugosity patterns in the head of *Pheidole* major and minor workers. (*) denotes that the frequency of rugosity pattern differs between worker type, based on a chi-squared pos-hoc test with Bonferroni correction (Table S3).

**
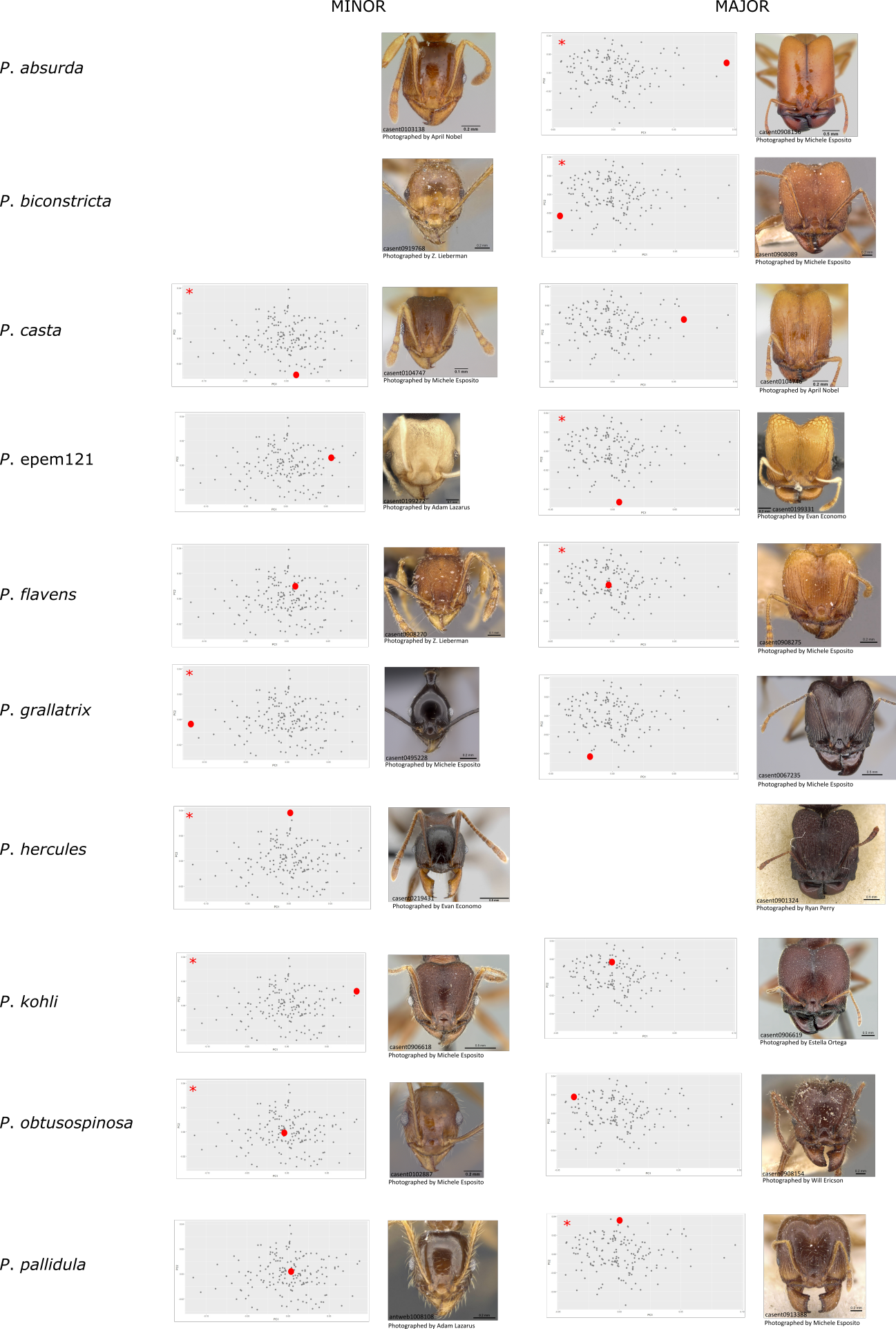
**

**Fig.S3.** Position in the morphospace of *Pheidole* major and minor worker head planar shapes according to Casadei-Ferreira et al. (2022). Red asterisks indicate the workers considered for FEA. Head full-face images were downloaded from AntWeb (AntWeb 2021). Three workers were not considered by Casadei-Ferreira et al. (2022) to the development of the morphospaces, namely the minor workers of *P*. *absurda* and *P*. *biconstricta*, and the major worker of *P*. *hercules*, therefore we display here only its heads’ pictures.

**Table S1.** Correlations between stress intervals and the two first dimensions of the PCA (Fig.3 on the main text).

| **Stress interval** | **Dim.1** | **Dim.2** |
| --- | --- | --- |
| V1 | -0.966 | 0.0818 |
| V2 | 0.864 | -0.314 |
| V3 | 0.942 | -0.257 |
| V4 | 0.953 | -0.140 |
| V5 | 0.959 | -0.126 |
| V6 | 0.979 | 0.060 |
| V7 | 0.957 | 0.202 |
| V8 | 0.866 | 0.437 |
| V9 | 0.557 | 0.783 |
| V10 | 0.078 | 0.925 |
| V11 | 0.247 | 0.949 |
| V12 | 0.695 | 0.380 |
| V13 | 0.768 | -0.167 |
| V14 | 0.634 | -0.598 |
| V15 | 0.432 | -0.6583 |

**Table S2.** Results of *post hoc* dunn tests with bonferroni corrections for repeated tests for the difference in stress magnitude between each pair of *Pheidole* species simulations.

|  |
| --- |
|  |
|  |

| **Comparisons** | **chi2** | **Z** | **P.adjusted** |
| --- | --- | --- | --- |
| P.absurda - P.biconstricta | 10546.8878175826 | 73.292504921378 | 0 |
| P.absurda - P.casta | 10546.8878175826 | 59.6311714396493 | 0 |
| P.biconstricta - P.casta | 10546.8878175826 | -13.95134429 | 6.95E-43 |
| P.absurda - P.epem121 | 10546.8878175826 | 48.7965974457227 | 0 |
| P.biconstricta - P.epem121 | 10546.8878175826 | -24.29932989 | 4.48E-129 |
| P.casta - P.epem121 | 10546.8878175826 | -10.48117174 | 2.37E-24 |
| P.absurda - P.flavens | 10546.8878175826 | 68.4330846901254 | 0 |
| P.biconstricta - P.flavens | 10546.8878175826 | -4.642001503 | 7.76E-05 |
| P.casta - P.flavens | 10546.8878175826 | 9.24945792630671 | 5.08E-19 |
| P.epem121 - P.flavens | 10546.8878175826 | 19.598067641973 | 3.61E-84 |
| P.absurda - P.grallatrix | 10546.8878175826 | 38.162799600545 | 0 |
| P.biconstricta - P.grallatrix | 10546.8878175826 | -35.03496153 | 1.49E-267 |
| P.casta - P.grallatrix | 10546.8878175826 | -21.23991646 | 9.19E-99 |
| P.epem121 - P.grallatrix | 10546.8878175826 | -10.67319676 | 3.06E-25 |
| P.flavens - P.grallatrix | 10546.8878175826 | -30.29534442 | 2.97E-200 |
| P.absurda - P.hercules | 10546.8878175826 | 67.5896737390242 | 0 |
| P.biconstricta - P.hercules | 10546.8878175826 | -5.622929302 | 4.22E-07 |
| P.casta - P.hercules | 10546.8878175826 | 8.29094050440419 | 2.53E-15 |
| P.epem121 - P.hercules | 10546.8878175826 | 18.6651063748704 | 2.14E-76 |
| P.flavens - P.hercules | 10546.8878175826 | -0.96933242 | 1 |
| P.grallatrix - P.hercules | 10546.8878175826 | 29.3812114366075 | 2.15E-188 |
| P.absurda - P.kohli | 10546.8878175826 | 86.3136460846668 | 0 |
| P.biconstricta - P.kohli | 10546.8878175826 | 12.4512596757759 | 3.10E-34 |
| P.casta - P.kohli | 10546.8878175826 | 26.561740402069 | 4.23E-154 |
| P.epem121 - P.kohli | 10546.8878175826 | 36.9044431888899 | 8.82E-297 |
| P.flavens - P.kohli | 10546.8878175826 | 17.0916461741207 | 3.85E-64 |
| P.grallatrix - P.kohli | 10546.8878175826 | 47.7413660052331 | 0 |
| P.hercules - P.kohli | 10546.8878175826 | 18.1039865672053 | 6.67E-72 |
| P.absurda - P.obtusospinosa | 10546.8878175826 | 52.718677127619 | 0 |
| P.biconstricta - P.obtusospinosa | 10546.8878175826 | -20.57496099 | 1.04E-92 |
| P.casta - P.obtusospinosa | 10546.8878175826 | -6.704715407 | 4.54E-10 |
| P.epem121 - P.obtusospinosa | 10546.8878175826 | 3.77986098251649 | 0.00353060876753995 |
| P.flavens - P.obtusospinosa | 10546.8878175826 | -15.87192569 | 2.23E-55 |
| P.grallatrix - P.obtusospinosa | 10546.8878175826 | 14.4871056938385 | 3.29E-46 |
| P.hercules - P.obtusospinosa | 10546.8878175826 | -14.92954127 | 4.76E-49 |
| P.kohli - P.obtusospinosa | 10546.8878175826 | -33.18620325 | 3.83E-240 |
| P.absurda - P.pallidula | 10546.8878175826 | 71.6709120721437 | 0 |
| P.biconstricta - P.pallidula | 10546.8878175826 | -1.181357841 | 1 |
| P.casta - P.pallidula | 10546.8878175826 | 12.6810336332328 | 1.69E-35 |
| P.epem121 - P.pallidula | 10546.8878175826 | 22.9760790683341 | 1.82E-115 |
| P.flavens - P.pallidula | 10546.8878175826 | 3.43642518954311 | 0.0132625150804262 |
| P.grallatrix - P.pallidula | 10546.8878175826 | 33.6453927906373 | 8.19E-247 |
| P.hercules - P.pallidula | 10546.8878175826 | 4.40919243442697 | 0.000233452798766626 |
| P.kohli - P.pallidula | 10546.8878175826 | -13.56588169 | 1.44E-40 |
| P.obtusospinosa - P.pallidula | 10546.8878175826 | 19.2701458888922 | 2.15E-81 |

**Table S3.** Results of a post-hoc test based on the Bonferroni correction for difference in the count of rugosity pattern between major and minor workers of *Pheidole*.

|  |
| --- |

| **Rugosity pattern** | **Variable** | **Major** | **Minor** |
| --- | --- | --- | --- |
| 0 | Count | 1 | 20 |
| 0 | Residuals | -4.29 | 4.29 |
| 0 | p values | 0.00014 | 0.00014 |
| 1 | Count | 23 | 57 |
| 1 | Residuals | -4.46 | 4.46 |
| 1 | p values | 6.50E-05 | 6.50E-05 |
| 2 | Count | 18 | 10 |
| 2 | Residuals | 1.61 | -1.61 |
| 2 | p values | 0.852286 | 0.852286 |
| 3 | Count | 97 | 53 |
| 3 | Residuals | 5.35 | -5.35 |
| 3 | p values | 1.00E-06 | 1.00E-06 |

| **Node** | **X coordinate** | **Y coordinate** | **Set** | **Mesh** | **Model** | **N nodes** | **N elements** | **Tresca** | **Error(%)** | | |
| --- | --- | --- | --- | --- | --- | --- | --- | --- | --- | --- | --- |
| 38650 | 0.006466 | 0.178906 | A | 2 | flavens | 81505 | 40488 | 2.50291 |  | | |
| 66807 | -2.82E-01 | -1.06E-01 | B | 2 | flavens | 81505 | 40488 | 2.39421 |  | | |
| 77019 | 2.79E-01 | -9.84E-02 | C | 2 | flavens | 81505 | 40488 | 2.16312 |  | | |
|  |  |  |  |  |  |  |  |  |  | | |
| 5384 | 0.007301 | 0.17675 | A | 1 | flavens | 164238 | 81745 | 2.47437 | 1.14 | | |
| 21627 | -2.81E-01 | -1.07E-01 | B | 1 | flavens | 164238 | 81745 | 2.43224 | 1.59 | | |
| 21777 | 2.78E-01 | -9.88E-02 | C | 1 | flavens | 164238 | 81745 | 2.17422 | 1.11 | | |
|  |  |  |  |  |  |  |  |  |  | | |
| 239695 | 7.55E-03 | 1.76E-01 | A | 3 | flavens | 328073 | 163504 | 2.45938 | 0.61 | | |
| 34118 | -2.82E-01 | -1.07E-01 | B | 3 | flavens | 328073 | 163504 | 2.4247 | 0.31 | | |
| 290906 | 2.79E-01 | -9.89E-02 | C | 3 | flavens | 328073 | 163504 | 2.19084 | 0.76 | | |
|  |  |  |  |  |  |  |  |  |  | | |
| 79525 | 2.73E-01 | 7.42E-02 | A | 2 | obtusospinosa | 83533 | 41504 | 2.02238 |  | | |
| 68221 | 6.99E-02 | -6.31E-02 | B | 2 | obtusospinosa | 83533 | 41504 | 2.98106 |  | | |
| 19984 | 4.46E-01 | -6.96E-02 | C | 2 | obtusospinosa | 83533 | 41504 | 2.94179 |  | | |
|  |  |  |  |  |  |  |  |  |  | | |
| 40586 | 0.272442 | 7.49E-02 | A | 1 | obtusospinosa | 166311 | 82784 | 2.01862 | 0.19 | | |
| 2836 | 6.91E-02 | -6.28E-02 | B | 1 | obtusospinosa | 166311 | 82784 | 2.99713 | 0.54 | | |
| 34077 | 4.46E-01 | -7.01E-02 | C | 1 | obtusospinosa | 166311 | 82784 | 2.95327 | 0.39 | | |
|  |  |  |  |  |  |  |  |  |  | | |
| 229159 | 2.73E-01 | 7.51E-02 | A | 3 | obtusospinosa | 326340 | 162645 | 2.00945 | 0.45 | | |
| 141380 | 6.97E-02 | -6.27E-02 | B | 3 | obtusospinosa | 326340 | 162645 | 2.98554 | 0.39 | | |
| 69249 | 4.46E-01 | -6.99E-02 | C | 3 | obtusospinosa | 326340 | 162645 | 2.96346 | 0.35 | | |
|  |  |  |  |  |  |  |  |  |  | | |
| 73486 | 1.285383 | 1.84E-01 | A | 2 | grallatrix | 82768 | 41115 | 2.95163 |  | | |
| 70125 | 1.099536 | -2.19E-02 | B | 2 | grallatrix | 82768 | 41115 | 3.89791 |  | | |
| 74260 | 1.410021 | -2.27E-02 | C | 2 | grallatrix | 82768 | 41115 | 3.60143 |  | | |
|  |  |  |  |  |  |  |  |  |  | | |
| 34910 | 1.272866 | 0.183519 | A | 1 | grallatrix | 165581 | 82408 | 2.91798 | 1.14 | | |
| 15149 | 1.096732 | -2.24E-02 | B | 1 | grallatrix | 165581 | 82408 | 3.9874 | 2.3 | | |
| 20364 | 1.414002 | -2.24E-02 | C | 1 | grallatrix | 165581 | 82408 | 3.66326 | 1.72 | | |
|  |  |  |  |  |  |  |  |  |  | | |
| 12680 | 1.271491 | 1.84E-01 | A | 3 | grallatrix | 329473 | 164198 | 2.91059 | 0.25 | | |
| 27178 | 1.096557 | -2.22E-02 | B | 3 | grallatrix | 329473 | 164198 | 4.01536 | 0.7 | | |
| 138209 | 1.414844 | -2.24E-02 | C | 3 | grallatrix | 329473 | 164198 | 3.6664 | 0.09 | | |
|  |  |  |  |  |  |  |  |  |  |  |  |

Table S4. Details of the mesh convergence tests to define the mesh density of plane head models. Node refers to the number of the node where Tresca values were collected. X and Y coordinates depict the location of the node in the mesh along the X and Y axis of orientation. Set represents the head region of each node (A = on the center of the head; B = near to left mandibular articulation; C = near to right mandibular articulation). Mesh codes for the mesh density (1 = mesh density as applied on the final FEA; 2 = the coarser mesh density; 3 = the refined mesh density). Model identifies the *Pheidole* species. N nodes and N elements depict the respective number of nodes and elements of the correspondent mesh. Tresca represents the stress value of each node based on Tresca failure criteria. % error is the variable considered to define mesh convergence, and is the result of the following equation: (previous - current mesh) / previous mesh x 100. We considered that convergence was achieved when the % error was less than 2% for all nodes analyzed, and the coarser mesh in the convergent pair was considered as the definitive mesh density.
