# Supplementary material for: Mechanical demands of bite in plane head shapes of ant (Hymenoptera: Formicidae) workers
Source: Ecol Evol. 2023 Jun 6;13(6):e10162. doi: 10.1002/ece3.10162 (PMC10244895; doi:10.1002/ece3.10162)
Supplement: Supplementary file 2 — Data S1 [file ECE3-13-e10162-s002.pdf]

Pheidole2D_FEA_submission_version

```
setwd("")

#Packages:

library(dplyr)

##
## Attaching package: 'dplyr'

## The following objects are masked from 'package:stats':
##
##     filter, lag

## The following objects are masked from 'package:base':
##
##     intersect, setdiff, setequal, union

library(ggplot2)
library(viridis)

## Carregando pacotes exigidos: viridisLite

library(tidyverse)

## -- Attaching packages --------------------------------------- tidyve
rse 1.3.1 --

## v tibble  3.1.6     v purrr   0.3.4
## v tidyr   1.2.0     v stringr 1.4.0
## v readr   2.1.2     v forcats 0.5.1

## -- Conflicts ------------------------------------------ tidyverse_co
nflicts() --
## x dplyr::filter() masks stats::filter()
## x dplyr::lag()    masks stats::lag()

library(hrbrthemes)

## NOTE: Either Arial Narrow or Roboto Condensed fonts are required to
use these themes.

##       Please use hrbrthemes::import_roboto_condensed() to install Ro
boto Condensed and

##       if Arial Narrow is not on your system, please see https://bit.
ly/arialnarrow
```

```
library(FactoMineR)
library(factoextra)

## Welcome! Want to learn more? See two factoextra-related books at htt
ps://goo.gl/ve3WBa

library(ggpubr)

#STRESS:

#Loading stress data of each species/simulation:

absurda <- read.table("absurda_ele_total2.txt", header = T, sep = "")
absurda$species <- 'P.absurda'

########

biconstricta <- read.table("biconstricta_ele_total2.txt", header = T, s
ep = "")
biconstricta$species <- 'P.biconstricta'

#######

casta <- read.table("casta_ele_total2.txt", header = T, sep = "")
casta$species <- 'P.casta'

########

epem121 <- read.table("epem121_ele_total2.txt", header = T, sep = "")
epem121$species <- 'P.epem121'

#########

flavens <- read.table("flavens_ele_total2.txt", header = T, sep = "")
flavens$species <- 'P.flavens'

########

grallatrix <- read.table("grallatrix_ele_total2.txt", header = T, sep =
 "")
grallatrix$species <- 'P.grallatrix'

#######

hercules <- read.table("hercules_ele_total2.txt", header = T, sep = "")
hercules$species <- 'P.hercules'

############
```

```
kohli <- read.table("kohli_ele_total2.txt", header = T, sep = "")
kohli$species <- 'P.kohli'

######

obtusospinosa <- read.table("obtusospinosa_ele_total2.txt", header = T,
 sep = "")
obtusospinosa$species <- 'P.obtusospinosa'

#######

pallidula <- read.table("pallidula_ele_total2.txt", header = T, sep = "
")
pallidula$species <- 'P.pallidula'


species <- rbind(absurda,biconstricta,casta,epem121,flavens,grallatrix,
hercules,kohli,obtusospinosa,
                 pallidula)#Combine all data into a single dataframe


species%>%
  group_by(Element, species)%>%
  mutate(tresca = mean(S.Tresca))%>%
  filter(row_number()==1) ->
  species_element#mean of tresca value for each element and species fro
m integration points (3 for #each element)

species_element <- species_element[, -c(2:5)]#removing unnecessary colu
mns from the dataframe

list_species <- split(species_element, species_element$species)#split t
he dataframe again to add data #on element area

absurda2 <- list_species$P.absurda
biconstricta2 <- list_species$P.biconstricta
casta2 <- list_species$P.casta
epem121_2 <- list_species$P.epem121
flavens2 <- list_species$P.flavens
grallatrix2 <- list_species$P.grallatrix
hercules2 <- list_species$P.hercules
kohli2 <- list_species$P.kohli
obtusospinosa2 <- list_species$P.obtusospinosa
pallidula2 <- list_species$P.pallidula

#AREA:

#Uploading data on element area for each species/simulation:
```

```r
absurda_area <- read.table("P.absurda_volume2.csv", header = T, sep = ",
")
absurda2$area <- absurda_area$EVOL

biconstricta_area <- read.table("P.biconstricta_volume2.csv", header =
T, sep = ",")
biconstricta2$area <- biconstricta_area$EVOL

casta_area <- read.table("P.casta_volume2.csv", header = T, sep = ",")
casta2$area <- casta_area$EVOL

epem121_area <- read.table("P.epem121_volume2.csv", header = T, sep = ",
")
epem121_2$area <- epem121_area$EVOL

flavens_area <- read.table("P.flavens_volume2.csv", header = T, sep = ",
")
flavens2$area <- flavens_area$EVOL

grallatrix_area <- read.table("P.grallatrix_volume2.csv", header = T, s
ep = ",")
grallatrix2$area <- grallatrix_area$EVOL

hercules_area <- read.table("hercules_ele_total_volume2.txt", header =
T, sep = "")
hercules2$area <- hercules_area$EVOL

kohli_area <- read.table("P.kohli_volume2.csv", header = T, sep = ",")
kohli2$area <- kohli_area$EVOL

obtusospinosa_area <- read.table("P.obtusospinosa_volume2.csv", header
= T, sep = ",")
obtusospinosa2$area <- obtusospinosa_area$EVOL

pallidula_area <- read.table("P.pallidula_volume2.csv", header = T, sep
 = ",")
pallidula2$area <- pallidula_area$EVOL

species_area <- rbind(absurda2,biconstricta2,casta2,epem121_2,flavens2,
grallatrix2,hercules2,kohli2,
                obtusospinosa2, pallidula2)

#Removing highest stresses:

species_area%>%
  group_by(species)%>%
slice_min(tresca, prop = 0.98)->
  species_area2#remove the 2% highest stress value of each species
```

```r
species_area2 <- species_area2[,c(1,2,4,3)]#reordering the columns

#Proportional stress values for intervals method:

species_area2 %>%
  group_by(species) %>%
  mutate(Max = max(tresca)) ->
  species_area3#create a column with the maximum stress value for each
species

species_area3$prop.tresca <- species_area3$tresca/species_area3$Max#new
 stress variable -> proportion o maximum stress value

species_area3 <- species_area3[,-c(4,5)]#removing unnecessary columns f
rom the dataframe

#Export simulation's data in the correct format to proceed with the int
ervals method:

species_tresca_split <- split(species_area3, species_area3$species) # l
ist of dataframes for each species and simulation type

species_tresca_split2 <- lapply(species_tresca_split, function(x) x[!(n
ames(x) %in% "species")])


# use numbers as file names
lapply(names(species_tresca_split2),
       function(x){write.csv(species_tresca_split2[[x]], paste0(x,"_XXX
X.csv"),#add any identification for each file
                             row.names = FALSE)})

## [[1]]
## NULL
##
## [[2]]
## NULL
##
## [[3]]
## NULL
##
## [[4]]
## NULL
##
## [[5]]
## NULL
##
## [[6]]
## NULL
```

```
##
## [[7]]
## NULL
##
## [[8]]
## NULL
##
## [[9]]
## NULL
##
## [[10]]
## NULL

#Intervals method:

#Generate the intervals:

FTupper = 0.69#upper threshold (see main text for details)

# Number of intervals: NIntervals

NIntervals = 5#repeat for each interval

# 2) Read the data.
# The data must be stored as .csv files in the same folder of the scrip
t
# Each .csv file must contain three rows with: 1) the number of the ele
ment,
# 2) area/volume of the element and 3) von mises stress, respectively.

file.name = list.files(pattern="*_XXXX.csv")#as defined in line 190
NFiles = length(file.name)

# 3) Create the matrix of intervals
# Each row with the area percentage for each interval and each file of
the matrix
# with the different models included

data.intervals = matrix(ncol = NIntervals, nrow = NFiles)

for (f in 1:NFiles) {

  data.values = data.matrix(read.csv(file.name[f], header = TRUE, sep =
 ","));

  # Get the number of mesh elements of the model

  NElements = nrow(data.values);
```

```r
# Create the internal matrix to store the intervals and other data

Counter.matrix = matrix(0,ncol = NIntervals, nrow = 5);

# Compute the range values for each interval (Tlower and Tupper)

Range.values = seq(0, FTupper, by=FTupper/(NIntervals-1));

# Start the Loop

for (i in 1:NElements) {
  for (j in 1:NIntervals) {
    if (j == 1){
      if (data.values[i,3] <= Range.values[j+1])
      {
        Counter.matrix[2,j]=Counter.matrix[2,j]+1;
        Counter.matrix[4,j]=Counter.matrix[4,j]+data.values[i,2];
      }
    }
    else if (j > 1 & j < NIntervals){
      if (data.values[i,3] > Range.values[j] & data.values[i,3] <= Ra
nge.values[j+1])
      {
        Counter.matrix[2,j]=Counter.matrix[2,j]+1;
        Counter.matrix[4,j]=Counter.matrix[4,j]+data.values[i,2];
      }
    }
    else if (j == NIntervals){
      if (data.values[i,3] > Range.values[j])
      {
        Counter.matrix[2,j]=Counter.matrix[2,j]+1;
        Counter.matrix[4,j]=Counter.matrix[4,j]+data.values[i,2];
      }
    }
  }
}

# End of the Loop

# Compute the percentage in each interval with respect  to the total
area

for (i in 1:NIntervals) {
  Counter.matrix[1,i]=Range.values[i];
  Counter.matrix[3,i]=100*Counter.matrix[2,i]/NElements;
  Counter.matrix[5,i]=100*Counter.matrix[4,i]/sum(data.values[,2]);
}

# Store the vector of intervals for the model f in the matrix of inte
```

```
rvals

  data.intervals[f,]=Counter.matrix[5,];

}

data.intervals=as.data.frame(data.intervals);
row.names(data.intervals)=file.name;

# 4) End of the script: save data

write.csv(data.intervals,'test5.csv')

#OBS.: This code was generated by Marcé-Nogué et al. (2017). See main m
anuscript for reference and #further details.

#Convergence of R² to define the final number of intervals:

# 1) Read all the files included in the convergence

intervals.data.5 = read.csv("matrix-of-5-intervals.csv",row.names=1, he
ader = TRUE, sep = ",")
intervals.data.15 = read.csv("matrix-of-15-intervals.csv", row.names=1,
header = TRUE, sep = ",")
intervals.data.25 = read.csv("test25.csv",row.names=1, header = TRUE, s
ep = ",")
intervals.data.50 = read.csv("matrix-of-50-intervals.csv", row.names=1,
header = TRUE, sep = ",")
intervals.data.75 = read.csv("matrix-of-75-intervals.csv", row.names=1,
header = TRUE, sep = ",")

# 2) Compute PCA for each case

PCA.5 = prcomp(intervals.data.5[,1:5], scale=T)
PCA.15 = prcomp(intervals.data.15[,1:15], scale=T)
PCA.25 = prcomp(intervals.data.25[,1:25], scale=T)
PCA.50 = prcomp(intervals.data.50[,1:50], scale=T)
PCA.75 = prcomp(intervals.data.75[,1:75], scale=T)

# 3) Calculate te R-squared values for convergence procedure

# PC1 convergence:

Rvalues.pc1 = c(summary(lm(PCA.5$x[,1]~PCA.15$x[,1]))$r.squared,
                summary(lm(PCA.15$x[,1]~PCA.25$x[,1]))$r.squared,
                summary(lm(PCA.25$x[,1]~PCA.50$x[,1]))$r.squared,
                summary(lm(PCA.50$x[,1]~PCA.75$x[,1]))$r.squared)

# PC2 convergence:
```

```r
Rvalues.pc2 = c(summary(lm(PCA.5$x[,2]~PCA.15$x[,2]))$r.squared,
                summary(lm(PCA.15$x[,2]~PCA.25$x[,2]))$r.squared,
                summary(lm(PCA.25$x[,2]~PCA.50$x[,2]))$r.squared,
                summary(lm(PCA.50$x[,2]~PCA.75$x[,2]))$r.squared)

# Table with results: R-squared values

data.pca = data.frame(Rvalues.pc1,Rvalues.pc2)
names(data.pca) = c("PC1","PC2")
rownames(data.pca) = c("PCA 5 vs. PCA 15","PCA 15 vs. PCA 25", "PCA 25
vs. PCA 50","PCA 50 vs. PCA 75")

# 4) PLot the PCAs and the R-squared values

par(mfrow = c(3,2))
plot(PCA.5$x[,1], PCA.5$x[,2], pch=19, cex=1.5, xlab = "", ylab ="", as
p=T, main = "B) 5 intervals", xlim=rev(range(PCA.5$x[,1])) , ylim = (ra
nge(PCA.5$x[,2])))
plot(PCA.15$x[,1], PCA.15$x[,2], pch=19, cex=1.5, xlab = "", ylab ="",
asp=T, main = "B) 15 intervals", xlim=rev(range(PCA.15$x[,1])) , ylim =
 rev(range(PCA.15$x[,2])))
plot(PCA.25$x[,1], PCA.25$x[,2], pch=19, cex=1.5, xlab = "", ylab ="",
asp=T, main = "C) 25 intervals", xlim=rev(range(PCA.25$x[,1])) , ylim =
 rev(range(PCA.25$x[,2])))
plot(PCA.50$x[,1], PCA.50$x[,2], pch=19, cex=1.5, xlab = "", ylab ="",
asp=T, main = "D) 50 intervals", xlim=rev(range(PCA.50$x[,1])) , ylim =
 rev(range(PCA.50$x[,2])))
plot(PCA.75$x[,1], PCA.75$x[,2], pch=19, cex=1.5, xlab = "", ylab ="",
asp=T, main = "E) 75 intervals", xlim=rev(range(PCA.75$x[,1])) , ylim =
 rev(range(PCA.75$x[,2])))
#dev.off()

plot(0,0,main = "F) Convergence", ylim = c(0, 1.2), xlim = c(1, 5))
points(Rvalues.pc1, col = c("tomato"), pch=19, cex=1.5)
points(Rvalues.pc2, col = c("salmon1"), pch=19, cex=1.5)
lines(Rvalues.pc1,lwd=1, col = c("tomato"))
lines(Rvalues.pc2,lwd=1, col = c("salmon1"))
abline(h=1, lty=3)
legend(4.3,0.5, legend= names(data.pca), pch=19,  col=c("salmon1","toma
to"),cex=1)
```

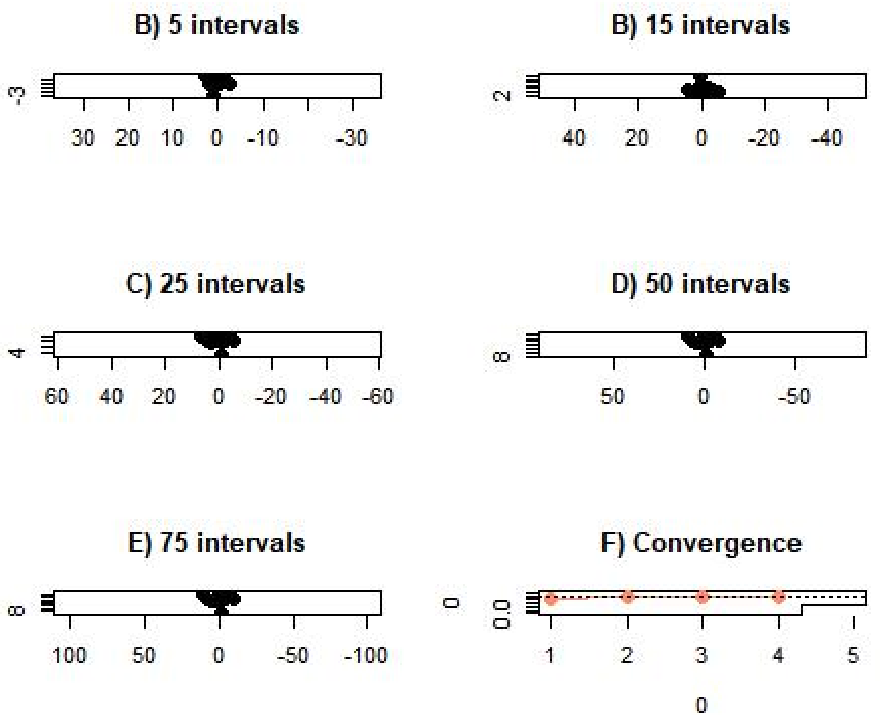


```
#OBS.: This code was generated by Marcé-Nogué et al. (2017). See main m
anuscript for reference and #further details.

#Final PCA:

stress.distrib = read.csv("test15.csv", row.names=1, header = TRUE, sep
 = ",")# "matrix-of-15-intervals.csv" is the paper version
row.names(stress.distrib) <- c("P. absurda", "P. biconstricta", "P. cas
ta", "P. epem121", "P. flavens",
                               "P. grallatrix", "P. hercules", "P. kohl
i", "P. obtusospinosa", "P. pallidula")

# 1) Multivariate analysis PCA

col.number = ncol(stress.distrib)
PCA.stress <- PCA(stress.distrib[,1:col.number],  graph = FALSE)

# 2) Define the parameters and create the biplot

# colors by group
group.colors = row.names(stress.distrib)

#colors by variable
interval.number = nrow(PCA.stress$var$coord)
interval.vector = seq(from = 1, to = interval.number, by=1 )
interval.colors = interval.vector
```

```
interval.palette = c("blue","cyan","green","chartreuse","yellow","gold",
"orange","red")

# Biplot: variables coloured by contribution to PCs
fviz_pca_biplot(PCA.stress,
                axes = c(1, 2),
                mean.point=F,                         #
                axes.linetype = "solid",

                # Fill individuals by groups
                geom.ind=c("point", "text"),
                pointshape = 21,
                pointsize = 5,
                col.ind= "black",                     #
                # fill.ind = group.colors,
                fill.ind = "black",
                alpha.ind = 1,                        #

                # Color variable by intervals
                geom.var = "arrow",
                col.var = interval.colors,
                arrowsize = 0.5,

                repel = TRUE) +                       # Avoid label overplo
tting


  #fill_palette(group.palette) +
  gradient_color(interval.palette)+
  labs(x = "PC1(%)", y = "PC2(%)")+


  theme(
    panel.grid.major = element_blank(),
    panel.grid.minor = element_blank(),
  )
```

```
#OBS.: This code was generated by Marcé-Nogué et al. (2017). See main m
anuscript for reference and #further details.
```
